# Implementing Community-based Health Planning and Services in impoverished urban communities: health workers’ perspective

**DOI:** 10.1186/s12913-018-3005-1

**Published:** 2018-03-20

**Authors:** Adanna Uloaku Nwameme, Philip Teg-Nefaah Tabong, Philip Baba Adongo

**Affiliations:** School of Public Health, University of Ghana, P O Box LG 13, Legon, Accra, Ghana

**Keywords:** Community-Based Health Planning and Services (CHPS), Urban health, Health policy, Health workers, Volunteers, Ghana

## Abstract

**Background:**

Three-quarters of sub-Saharan Africa’s urban population currently live under slum conditions making them susceptible to ill health and diseases. Ghana characterizes the situation in many developing countries where the urban poor have become a group much afflicted by complex health problems associated with their living conditions, and the intra-city inequity between them and the more privileged urban dwellers with respect to health care accessibility. Adopting Ghana’s rural Community-Based Health Planning and Service (CHPS) programme in urban areas is challenging due to the differences in social networks and health challenges thus making modifications necessary. The Community Health Officers (CHOs) and their supervisors are the frontline providers of health in the community and there is a need to analyze and document the health sector response to urban CHPS.

**Methods:**

The study was solely qualitative and 19 in-depth interviews were conducted with all the CHOs and key health sector individuals in supervisory/coordinating positions working in urban CHPS zones to elicit relevant issues concerning urban CHPS implementation. Thematic content data analysis was done using the NVivo 7 software.

**Results:**

Findings from this appraisal suggest that the implementation of this urban concept of the CHPS programme has been well undertaken by the health personnel involved in the process despite the challenges that they face in executing their duties. Several issues came to light including the lack of first aid drugs, as well as the need for the Integrated Management of Neonatal and Childhood Illnesses (IMNCI) programme and more indepth training for CHOs. In addition, the need to provide incentives for the volunteers and Community Health Committee members to sustain their motivation and the CHOs’ apprehensions with regards to furthering their education and progression in their careers were key concerns raised.

**Conclusion:**

The establishment of the CHPS concept in the urban environment albeit challenging has been fraught with several opportunities to introduce innovations which tailor the rural milestones to meet urban needs. Modifications such as adjusting timing of home visits and renting accommodation in the communities for the CHOs have been beneficial to the programme.

**Electronic supplementary material:**

The online version of this article (10.1186/s12913-018-3005-1) contains supplementary material, which is available to authorized users.

## Background

Population growth is staggering worldwide, with region-specific population-related concerns. The United Nations Population Fund postulated in 2007 that the urban populations in Africa will double by 2030 with the fastest growing group being the urban poor [1]. In addition, 72% of Sub-Saharan Africa’s urban population currently lives under slum conditions making them susceptible to ill health and diseases. In Ghana, the population has increased from 6.7 million in 1960 to about 25 million in 2010 with the urban population being 50.9% of the total. With a national growth rate of 2.5%, the country has an urban growth rate of 4.2% and a much lower rural growth rate of 1.0% [2].

Similar to other developing countries, Ghana’s fastest population growth takes place in urban areas which leads to the formation of informal peri-urban settlements and slum communities that lack basic facilities such as sanitation, clean water sources, or healthcare facilities. In these communities, a health crisis has emerged, and in addition to poor referral systems for maternal and child health emergencies, there is an increase in infant and child mortality rates, rapid spread of HIV and other STDs, unwanted teenage pregnancy and unsafe abortions [1]. The urban poor have thus become a group much afflicted by complex health problems associated with the living conditions in their communities, and the intra-city inequity between them and the more privileged urban dwellers with respect to health care accessibility [3, 4]. The low socio-economic status and poor education of this group therefore, makes it imperative for service delivery to be provided at household and community levels.

Community-based health interventions systems that are closely coordinated with traditional leaders and communication networks have proven to be effective methods of health care delivery in rural Ghana [5]. In an effort to increase access to primary health care at the community level, a three village pilot was carried out in the Upper East Region by the Navrongo Health Research Centre in 1994 to map out culturally appropriate strategies for the implementation of the Community-based Health Planning and Services (CHPS) programme [6]. The success recorded by this pilot project led to a nationwide programme being launched in 2000.

Built on six implementation milestones, the objective of CHPS is to reverse the trend of grim maternal and child health indicators particularly in the underserved communities of the rural districts. These milestones include preliminary planning, community entry, creation of community health compounds, posting of Community Health Officers (CHOs) to these compounds, procurement of logistics and deployment of volunteers [7]. Throughout these stages, a partnership is developed with the community leaders and members in a bid to come to a general consensus on the needs of the community thereby forging a feeling of ownership of the initiative within the community. This perception of ownership is the driving force behind the success of the CHPS initiative and the CHOs must continually educate and motivate the community to ensure their sustained interest in the programme [7]. Services provided by the CHOs include household visits for antenatal care, family planning services, and health education; outreach clinics for provision of child welfare services; and school health services. In-service training workshops organised for CHOs serve to improve basic clinical and midwifery services and develop diplomacy and counselling techniques [7].

As this strategy continues to promise better access and utilization of family planning services in numerous parts of the country, the question arises if such a health policy, with all its community-centered approaches, has a place in the urban areas of the country. The urban setting presents immense challenges that put urban CHPS implementation at variance with the model originally applied in the rural settings. Many migrants in the study area arrive to dwell as “squatters” in informal settlements and this flux is a result of rural dwellers migrating to urban areas in search of economic opportunities in a bid to lift themselves and their families out of poverty. Implementation of the CHPS model in the Ga East Municipal, albeit challenging has presented an extraordinary opportunity to create innovative strategies to address the provision of a health delivery system within this special population of urban dwellers [8].

Urban CHPS seeks to apply alternative strategies of the six milestones of rural CHPS to suit its own environment. The differences between rural and urban areas with regards to social networks and health challenges make these modifications necessary in order to ensure that quality basic health services are made available to these disadvantaged urban communities. The Community Health Officers and their supervisors are the frontline providers of health in the communities and in view of the increasing need for an adequate health sector response to the growing problems in such peri-urban areas, as well as the special circumstances which have made the implementation of urban CHPS challenging, there is a need to analyze and document the health sector response to urban CHPS.

This study will report on a qualitative appraisal of reactions of the health care personnel (CHOs and their supervisors) towards the implementation of CHPS in an urban informal settlement of Accra as a means to inform further modifications and add to a database of literature on urban health in the growing cities of Africa’s developing nations.

## Methods

This qualitative appraisal formed part of a quasi-experiment that was run to test proven health innovations in Ghana (Ghana Essential Health Intervention Programme, GEHIP) and Tanzania (Tanzania Essential Health Intervention Programme, TEHIP) on maternal and child health [9]. In Ghana, urban CHPS was piloted in the Ga East Municipal in 2011 beginning with two zones and has currently been scaled up to 15 operational zones to cater for a total population of 30,633 people. The Ga East Municipal is located in the northern part of Greater Accra Region. It has a population of 259,668 people and covers an area of 166 km^2^ [10]. The population is concentrated mainly along the urban and peri-urban areas of its four sub-municipals namely, Dome, Taifa, Haatso and Abokobi. Over the past two decades some areas of the Greater Accra Region has had a rapid increase in population growth with the concomitant spatial expansion into peri-urban surroundings and Ga East Municipal in the region is an area experiencing this fast population explosion [11]. The economic activities prevalent in the municipality are commerce, agriculture, service and industry though a large part of the working force have no employment, highlighting the high poverty level prevailing in the municipal [12].

### Data collection and analysis

The study was carried out using qualitative research methods to explore perspectives regarding the implementation of Community-based Health Planning and Services in poor urban areas among healthcare personnel. Researchers conducted a total of nineteen (19) in-depth interviews (IDIs) using in-depth interview guides for CHOs [see Additional file 1] and supervisors [see Additional file 2] to explore the relevant issues with regards to implementing CHPS in a urban environment, challenges faced and proffer solutions to these drawbacks. All the participants were chosen purposively and were recruited by phone calls. Fourteen interviews were held with CHOs working in urban CHPS zones as the CHOs are the frontline workforce with regards to CHPS implementation therefore their experience on the field offers a wealth of knowledge in mapping out the way forward for the programme. The interview guides were designed to probe and elicit relevant issues concerning urban CHPS implementation, the challenges CHOs face while carrying out their day-to-day activities, as well as proffered solutions to these drawbacks.

To further explore the issues concerning urban CHPS implementation, five IDIs were conducted with key health sector individuals in supervisory/coordinating positions as their wealth of experience concerning the progress of the project will be brought to bear in decisions taken towards charting the way forward for the project. Reports from community based activities are submitted by the supervisors to the district health management team and is then sent to the regional and national levels. Information about the participants is summarized in Table 1 below:

**Table 1:** IDI study participants

Participants	Number interviewed	Sex	Educational background
Community Health Officers	14	All female	Attended 2 years of Community Health Training School
Community Health Supervisor	1	Female	Attended 2 years of Community Health Training School
Public Health Nurses	3	All female	Attended School of Nursing for 3 years, I year midwifery and 18 months of Public Health Nurses’ Training School
District Health Director	1	Female	Has an MBBS, and MPH

The IDIs were conducted by two field research officers who were Masters in Public Health degree holders. These researchers were trained by the Principal Investigator. The data collection was done over a period of 1 month and the face-to-face interviews were held in various health posts and offices in the district. The IDIs were conducted in English Language and the interviewers obtained written consents from the participants to audio record them with each interview lasting between 30 to 60 min. The researchers then transcribed the information elicited whilst keeping the identities of the participants confidential. The transcripts were further double checked to ensure absence of errors and the data was analysed using the Nvivo 7 software and the thematic content analysis technique. A codebook was developed by the research team and the various codes were created as nodes within the software. The transcripts were then imported into NVivo, read line-by-line and pertinent statements made by respondents were coded unto the relevant nodes. As the coding progressed, relationships between the coded segments developed and themes were formed. The coding was done by two analysts and results were compared as they proceeded. Where there were any differences in opinion, a third party was invited to weigh in on the subject. The emerging themes were then used to narrate the results supported with illustrative quotes from respondents.

## Results

During the interviews, various aspects of the implementation of the CHPS programme in urban areas were highlighted. These include the health workers’ knowledge about the urban CHPS concept, the services offered by CHOs during home visits, the community acceptance of the urban CHPS concept, the welfare of the CHOs, supervision and provision of logistics for the programme, and community volunteerism.

### Understanding the urban CHPS concept

The field training for the CHOs after graduation from the Community Health Nurses Training Schools is done in rural CHPS zones thus the CHOs were able to make a clear distinction between CHPS as practiced in the rural areas, and that which is being implemented in the urban area:

*...in the urban one, it does not get the people in the community normally compared to the rural area that they always need you. They always need you - they don’t have any nurse around, any doctor around. Anytime when they see you, they want to share their problem with you. But with the urban area you can see that there is this health facility here, another TBA here, let’s say a private clinic there. So anytime he’s having any problem, he’ll go to that place but when you go there, you have to carry it to them, know how to talk to them even for you to get closer to them to share with them… (IDI, CHO)*

Catering for an urban population has required the adaptation of a new set of service operations tailored towards their peculiar needs. Having been in the urban CHPS zones for 2 years, with the consequent adaptations to these new set of keystones, the CHOs were able to assess the progress of the initiative in their various communities.

*[It's] about 49% successful…you know in the urban set-up, most people are busy and it’s not everybody that is being covered now as we are speaking, so the few people who really accept us when we get there, that’s why I said it’s about 49%. (IDI, CHO)*

### Home visiting and service delivery

The CHOs deliver a wide range of services at the homes of their clients and these include immunization, antenatal and postnatal care, health education, family planning, referral of severe disease conditions, as well as school health visits. Delivery of these services to an urban population is tailored to the needs of the individuals in the communities who are met during the home visits. As the CHOs explained:

*… so you can enter into a house and you’ll have about 15 households, inmates in the house. You can get an aged there, you can get a baby, a pregnant woman- you’ll get all your targeted population there. So when you get there, if there is a pregnant woman you take care of the pregnant woman, if there is a baby you take the weighing card to see if the child is due for immunization or the child is due for a weighing. So upon finding whatever the person is due for, you do it for the person. (IDI, CHO)*

*…it depends on the group of people you meet. When we meet the nursing mothers like this, most of them complain of their children not eating and stuff, so the nutrition when you come there, they are interested in that one. And when you get to the aged, because they are also having problem with their knees and stuff, we talk to them about their diet and things they should do to keep fit, and so they are interested in that. The adolescents too- some of them- the family planning services, they want to know more. (IDI, CHO)*

The CHOs are expected to achieve a home visiting quota of 10 households per day but this is not always possible given the fact that urban dwellers are more likely to leave home early in the day in pursuit of a living. This has been a challenge that the CHOs and their supervisors have had to make modifications such as rescheduling of visit times to address.

*…when you go into the community, you look at the dynamics of that community. At the end of the day, the strategy is home visit so they have to modify their operational times to suit what goes on in the community. (IDI, Supervisor)*

*The only challenge they have is at times they will go there and they won’t meet them. They will go to the homes and they won’t meet them so they reschedule their visits. Instead of going there in the morning, they go there at the weekend because most of the clients leave early in the morning for their work. Most of them are traders and some of them work in Accra so if you don’t leave early, you miss a lot so they’ll go there they don’t meet them. They go there in the evening…at first it was only in the morning, so we rescheduled and go there in the evening and on the weekends too and I think that is working for them. (IDI, Supervisor)*

In addition to home visiting rescheduling, the CHOs have also had to restrategize some of their service delivery operations, such as setting up Child Welfare Clinics within the communities to suit the preferences of the mothers in the communities.

*With the child welfare, they come across defaulters. If you ask them to go to the main stream child welfare clinics, they don’t go so at times, what they do is, they take them there. But now they have the child welfare within their catchment area so the mothers come there and they go there to do the child welfare. They don’t want the CHOs to come into their homes because they collect money from their husbands that they are going to the CWC so if you come to their home, then there will be no money for them but within the catchment area there they have a place where they will meet and do the CWC for them. (IDI, Supervisor)*

The CHOs however felt that their inability to provide some specific services such as long term family planning, malaria Rapid Diagnostic Testing, and first aid may lead to customer dissatisfaction amongst the community members.

*One challenge with the family planning is, now our people have understood the long term methods and some are willing to but when we talk to them and they accept the method we have to refer them to either Taifa or Madina. Because we are referring them they come back to choose the short term that we will do there. So it looks like they will appreciate it if we’re the one delivering that long term to them because when we want to refer them they will say, “Ok I’ll think about it.” (IDI, CHO)*

*Yes, like with this malaria- if we can have the kit then when you go and somebody is giving a complaint and signs and symptoms are likely to be malaria, at least if you check for the person and it’s positive...I think the people would feel satisfied better than just listening to the signs and symptoms and saying go to the hospital. So they feel like you’ve done something for them. (IDI, CHO)*

### Community acceptance of urban CHPS

The reactions of members of the community to the CHPS programme have been mixed. The CHOs pointed out the varying experiences they have encountered in their different zones. While most of the CHOs were of the opinion that the community members were appreciative of their work, they also pointed out the fact that there were still individuals who were not yet fully supportive of them.

*Most of them appreciate our work, but few of them do not appreciate our work. Sometimes we will be moving out and they will say “Maame nurse, I have a headache” [direct translation from Twi]; “Maame nurse, come and do this for me.” So they appreciate our work, it’s just a few of them who do not appreciate our work. (IDI, CHO)*

*For my community, I would say that they are good. I don’t have any problems with them because, even if you are passing and you do not go to the other house, somebody will tell you that the other person is there so that we will go. So they all know us and if they see us we don’t have any problem with them. (IDI, CHO)*

*I for one, I will talk for myself. I feel welcomed. Most of them give us a very warm reception every time we go into the community and then those people are people that I think know what we are doing, and they know the benefits or they understand the CHPS concept well. And, I am saying this because most of the people in the community as at now do not understand the concept of the CHPS. (IDI, CHO)*

According to the CHOs, the way in which the urban CHPS programme is perceived in the community differs along gender, age, and educational lines with the women, the aged and the poorly educated showing the most interest in the CHPS activities.

*For the women they accept us, but for the men, sometimes they don’t understand why we are moving from house to house (meanwhile they have a sickness). But the women, because they have children under 5 and family planning services rendered to them, they are always welcoming us. But the men are not welcoming, except for the aged. Because we take their BP, they are very welcoming. (IDI, CHO)*

*Oh and there is this other problem, you know they see us to be children. They think we are too small because most of us look so smallish, they think we are children, they don’t see the reason why they should sit down and listen for us children to come and dictate to them what to do. (IDI, CHO)*

*When you go to the house and you see that this woman is educated, the way the person relates to you is different from a woman who is not educated. That is because sometimes those who are educated feel they know and those who are not educated, they listen to whatever you will say. But those who have gone to school, sometimes they feel they know so when you’re talking to them, they make themselves some way. (IDI, CHO)*

### Welfare of the Community Health Officers

Implementing CHPS in an urban setting poses a huge challenge with regards to accommodation for the Community Health Officers due to the lack of communal land space. This leads to some CHOs having to render services while living outside their catchment areas. This arrangement has its attendant problems as explained by some of the CHOs.

*Ok, with the CHPS concept, what we know is you normally live at the CHPS compound, that is for the rural one. But since this one is urban CHPS, you come from your house to your work place but it would have been more effective if we were living within the community in which we are working but since it’s not like that, sometimes when you’re coming you’re supposed to get to work at 8 but transportation issues here and there, you could get there at 8.30 before you settle down, pack your things and move to the houses. (IDI, CHO)*

*With me per se, sometimes you get home and the people of the community will call you and say there is a problem so you have to stop whatever you are doing and come back into the community and transportation wise too, it creates a problem. (IDI, CHO)*

A few of the CHOs who had been fortunate to secure accommodation within their zones were able to point out the advantage of being able to render services to community members outside of regular working hours.

*Yes, yes, some of them when you go you don’t go and meet them, but because we are in the community in the evening you can go there. Sometimes when you want to immunize a child some of them are not around, most of them go to work early in the morning so in the evening we can go. And even when you are at home, they come for family planning. Last time I was there at even 9 o’clock - a woman called me, so I had to leave my bed and go and do it for her, so I think it’s good to be there. (IDI, CHO)*

*Because now my [work] partner is living here - she was also living far away, but because of the house here, she has moved in - so the community people they have come to know that, yes she is living here now so at times they come for evening home visits. So at times maybe I will take up the morning one then, in the evening she will come round and visit places that I have been able to go but I didn’t meet the mother there. (IDI, CHO)*

The paucity of accommodation in these areas led to the CHOs being required to share any accommodation that was secured and some of them did not appreciate this living arrangement.

*No, the place they got for us- the room is small it can't contain the two of us and me like this, I need privacy. As we are, one wouldn’t like to share a room with your [work] partner that much so me I’m still residing at where I rented my apartment so I don’t stay in the house they’ve rented. (IDI, CHO)*

One of the supervisors suggested that renting of offices for the CHOs instead of living areas may be a solution to challenges concerning accommodation.

*...maybe what we can do instead of renting accommodation for nurses, rather we should open more offices. (IDI, Supervisor)*

Moving about in the communities also poses several difficulties for the CHOs such as poorly constructed drainage systems, rugged topography, unpredictable weather conditions and high temperatures, as well as unfriendly domestic animals.

*Sometimes when you’re moving around the community, you meet certain hazards. There are big gutters you have to cross to the other side. Sometimes, you’ll have to walk through the big gutter to the other side. Sometimes too, some parts don’t have a bridge so if you want to go and pass through where it has a bridge, you’ll have to go to a longer place before you get to where you want to do your visit so they have put these stones in the big gutter that you’ll have to try and cross. That one too, it becomes very difficult and sometimes too, there are some houses that you’ll go that you’ll meet dogs. They all make it very difficult. (IDI, CHO)*

*The problem that we have, you know sometimes we walk in the sun for long then you are tired you’re sweating so you have to find a place to perch yourself, sit down somewhere and then relax for a while. (IDI, CHO)*

Whilst conducting home visits, the CHOs meet a variety of cases which they are expected to tackle using knowledge acquired from the training schools and field practical experience. A few of the CHOs found the training they had received adequate for carrying out their job, while the majority felt that they were lacking in some aspects of service delivery.

*Ok, the knowledge that I have is helping me to take care of people in their various homes - how to talk to them, how to relate in order to know their challenges about health. So the knowledge they are giving us is okay. (IDI, CHO)*

*Not really, because some of the challenges that we meet need more knowledge…there are some things you meet and you have to refer some of the problems and even some like our family planning services too - some people want the long term, but we haven’t been trained and we don’t know anything about it. We know, so when they come we only have the short things that is the pills and others. So when they come and they want something like that too we tell them to go to the facility, so we need more training. (IDI, CHO)*

The supervisors, however were of the opinion that the training given to the CHOs was adequate, and that the CHOs only required periodic refresher courses to update them on health issues that arise with time.

*I think for now it’s ok, because every now and then we update them, we refresh them. Apart from the training we have other programs that they are invited to and we do refresher clinic. Unlike the structured one, there are other ones that the region provides for CHOs and so I think there is enough. (IDI, Supervisor)*

### Supervision and logistics

Findings from the study show that the CHOs regard the supervision of their home visiting activities as inadequate. The supervisory activities are carried out by the CHPS coordinators at the district and regional levels and involves periodically accompanying the CHOs on home visits to supervise their activities within the community and offer support where needed. In a bid to discharge these duties, they face several challenges such as transportation difficulties, rough terrain and poor staff strength. Though the supervisors acknowledge that these challenges make for inefficient supervision, a few of them opined that they were able to find other avenues of ensuring that the work was done.

*The supervisor that we have, when she comes at times she likes to come with us to the field, but not always. (IDI, CHO)*

*It’s like the zones are not well laid out. Even if you want to take a vehicle to a point up and continue with the walking, that’s where it becomes difficult because you can't get through and when it rains, you can't do anything -the place becomes muddy and then it’s like when you meet your clients in the house is when you can do your work. It is when you don’t meet them that you can't work. (IDI, Supervisor)*

*Because I’m assigned to a different zone, sometimes Mondays like this we have school health services and I have to go to the CHPS too so sometimes I don’t go there because of the school health services that we have so it’s like I have some divided attention for them. (IDI, Supervisor)*

*The project really needs monitoring. In the whole clinic there is only one vehicle- that vehicle is for the medicine at Abokobi when we are short of vaccines. That one is for health insurance if they have to chase claims, that same vehicle is for pharmacy to go and bring medical stocks. It can go three times in a week, so monitoring is not effective and I tell them. Because the way I am firm, and I know how to get them to work, I do, and I use the phone to call. (IDI, Supervisor)*

While doing the home visit rounds, the CHOs carry their logistics in a home visiting bag that contains a weighing scale, a thermometer, a sphygmomanometer and stethoscope, a glucometer, contraceptives, vaccines and registers. Most of the CHOs and supervisors find these supplies inadequate due to the fact that occasionally the CHOs face situations which they are not equipped to handle.

*No, they are not adequate because sometimes you meet other cases that are not child welfare related, that are not diabetes related nor high blood pressure. It could be malaria or diarrhea and we don’t have even the first aid for diarrhea. (IDI, CHO)*

*We don’t have first aid drugs, they haven’t given us first aid drugs, so when you go and someone is having a headache we can't give out paracetamol and later refer the person. (IDI, CHO)*

*Their home visiting bag, the BP apparatus that they have, they share it between the two zones so if I am going with the BP apparatus maybe another time the second person too will take it. And the weighing scale, we also have one weighing scale which they share so if they can get enough items for them at least. If there will be paracetamol, maybe syrup paracetamol or tablet paracetamol- they get to a house, the person is feverish and they can give first aid before referring that may be better but in their home visiting bag, there is no first aid drug in the bag. (IDI, Supervisor)*

The supervisors were however able to point out the bottlenecks surrounding the dispensation of drugs by CHOs. The quandary of deciding what drugs could be dispensed by the CHOs, the financial implications, as well as the danger of incomplete treatment of patients were all concerns raised by them.

*That is- what medicines should be readily available, how and who is to account for them? Would they have to pay? We haven’t gotten to the bottom of this issue, but we say that for them to be more useful, that is the CHOs, there is a need for them to carry something as their credibility is at stake. (IDI, Supervisor)*

*It is first aid so you would give two tablets and not the whole thing, because when you give the whole thing to the person, you might think that is the end of it. If I give you paracetamol and the pain goes, the BP is still there, but they don’t go to hospital. So this one we have to really look at it and see how best to do whatever we do and find a way to sustain it. (IDI, Supervisor)*

### Community volunteerism

While a zone is being established, members of the communities are identified to function as volunteers and assist the CHOs in carrying out their duties. They aid in identification of health related cases in the community, and in tracing defaulters of essential health services such as antenatal care, family planning and child welfare clinics. Key members of the communities are also identified to serve as members of the Community Health Committee with their main roles being fund raising, arbitration and general sustenance of the programme as a community project.

*Under normal circumstances, what volunteers are supposed to do is, when we are going for home visits, since the community members know them, they have to go with us to the community so that when we go and there is any challenge concerning language or something like that, they will help us out. But it isn’t so at our place because the volunteers also happen to be working. (IDI, CHO)*

*And then with the committee, they are also helping us to talk to the community members because they also live in the community and know the community very well. Every time we have a problem, we go to them and we tell them maybe we had this problem somewhere then they also come in and then talk to them, and then because they know them they listen to them more than they listen to us. (IDI, CHO)*

Despite being informed of the voluntary nature of their assistance during their training sessions, participation by the volunteers and CHC members in CHPS activities and meetings has dwindled over time, largely due to the fact that there is no monetary compensation involved.

*The thing is like this- they are doing something that they are not being paid for so you know anything you do without money or something (how should I put it)…you’re not being paid for the services you are rendering, your commitment is not like how you’re being paid. So if it were to be that they were being paid, I think that they would have been more committed than doing that voluntary work. So they feel (that’s how I feel)… they feel that if the person has something to do which will benefit him, why should he come for a meeting that he will not get anything out of. (IDI, CHO)*

*Some of the CHCs they have other things doing so organizing meetings is a problem. We have teachers, we have pastors and the imam too, so the time you have you want to schedule the meeting but they are also having other things to do, so they will not be ready for you. And the day too that they might be ready, you might not be ready, maybe having another task to do. (IDI, CHO)*

*The volunteers are part of the CHCs, but for the past three months we are not meeting because when you call for the meeting, they don’t come only one person will come. So for the last few months they don’t come. (IDI, CHO)*

This situation has led to the dilemma of either working understaffed or continuously recruiting and training new volunteers as the need arises.

## Discussion

The implementation of the urban CHPS model is an innovative primary healthcare delivery model that applies lessons learned from implementation at the rural settings while making modifications to suit the impoverished urban settings. Findings from this appraisal suggest that the implementation of this urban concept of the CHPS programme has been well undertaken by the health personnel involved in the process despite the challenges that they face in executing their duties.

During the appraisal, several issues came to light regarding the progression of the urban CHPS concept with the provision of first aid drugs and the institution of the Integrated Management of Neonatal and Childhood Illnesses (IMNCI) programme being of the utmost importance. The CHOs were of the opinion that provision of these services will buttress their relevance in their communities. A study carried out in five urban sites in four African countries established that Community Case Management of malaria (CCMm) in urban areas is practicable and is viewed as a welcome development by urban populations [13]. Furthermore, the role played by community health workers in the reduction of inequities with regards to access to family planning services has been documented [14]. This sheds light on the challenges faced by CHOs who pointed out the need for long term family planning to be made available in the community as referring women to faraway facilities to access the service was proving ineffective due to non-compliance to referrals. Equipping the CHOs with the skills and logistics needed to cater to the needs of the community members can ensure that there is revenue generated internally to support the programme.

The need for the training of additional nurses, the organisation of more refresher courses for the CHOs, and better supervision of home visit acivities was reiterated by the CHOs and supervisors alike. This workforce capacity building will ensure better coverage of the communities mapped out while supervision and refresher courses will equip the CHOs with up-to-date skills they need to handle the cases they meet in the course of carrying out their duties. A study carried out in the Upper East Region of Ghana suggests that interventions geared towards addressing targeted health needs of the communities, as well as in-service refresher training for frontline workers, go a long way towards strengthening the service delivery available to the communities involved [9]. Furthermore, research conducted in Benin and Kenya highlighted the fact that proper supervision and attending training courses led to the health workers feeling better prepared and self-assured to do their jobs [15]. Training the CHOs in long term family planning services, and equipping them to manage childhood illnesses such as malaria and diarrhoea would improve the acceptance level of the programme amongst the community members, as well as give the CHOs a sense of pride in the services they offer. Gaining the trust of community members has been observed to boost the self-esteem of health workers and being able to provide basic health care services aids in validating the need for their services in the community [16].

The concept of volunteerism amongst the community members was almost non-existent due to the fact that the community members did not fully understand the CHPS concept. Furthermore, most of the residents had come from different tribes in the rural areas to settle in the urban areas in search of a better means of livelihood thus leading to a lack of homogeneity amongst members of the community. The fact that these communities do not have traditional leaders also led to the lack of communal bonding amongst them. The CHOs and their supervisors had resorted to soliciting the help of alternative influential members of these communities such as teachers, religious heads and market-place leaders. However, the need to provide incentives for the volunteers and CHC members to sustain their motivation to continue supporting the CHOs was a key concern expressed by the CHOs and their supervisors. Several studies have shown that remuneration for community volunteer workers can lead to undermining of relationships with community members, problems with sufficiency of the amount paid as well as with sustainability of the payment, and inequity amongst the workers as all the community workers are most of the time, not on the same salary scale [17, 18]. Conversely, other studies have documented that paying the community volunteer workers has been known to result in a decrease in attrition rates, increased quality of work done, built up economic equity and programme consistency [19, 20]. Non-monetary compensations for volunteers that have been suggested include recognition by the community, opportunities for further training, and provision of privileges such as access to credit through bank loans [21–23]. These approaches could be tailored to the urban environment using mobile phone credits as compensation, or provision of small-scale business capitals for the volunteers.

The CHOs further pointed out that even though durbars had been held at the inception of the programme, continuous education of the community members was required to ensure that they understood completely, the purpose of the CHPS concept and their role as community participants. Nyonator et al. [5] pointed out the possibility of community interest in the programme waning if the community was not kept continuously educated and motivated to participate in the programme. To this end, holding community durbars intermittently, as well as local radio broadcasts will aid in sensitizing the community members to the benefits of the programme. Even though the results allude to the fact that there are more health care providers in urban areas than are prevalent in rural communities, it has been demonstrated that there is a big gap between the quality of care available to the urban poor and that available to their wealthier, and perhaps rural, counterparts [24]. This buttresses the need to get the community members fully on board with the programme as a means of getting health care services to the less priviledged. Moreover, a health care delivery system that can be tailored to the rigours of urban living such as the need to leave the household early and return late, is a worthwhile initiative.

In addition to all the work-related concerns raised by the CHOs, they also expressed personal apprehensions with regards to accommodation, furthering their education and progressing in their careers. As posting CHOs to live in the communities is one of the milestones pivotal to the CHPS programme [7], and urban areas are devoid of communal land for this purpose, renting of accommodation as a CHPS base may be an option worth exploring in the urban context. Other studies have pinpointed areas of concern to the CHO’s welfare to include personal worries such as shortage of staff to cover for CHOs on leave, difficulties in advancing their careers and separation from their families leading to boredom and isolation [5, 25]. Providing opportunities for health workers to advance their careers has been shown to be a motivating factor for them [26]. An improved workforce will ensure that there is another CHO or a volunteer to fill the gap created whenever a CHO goes on a short leave to visit family members, or on a more prolonged study leave.

### Limitations of the study

Even though this study addressed an important gap in literature, there are a few limitations to be noted. The implementers of the intervention organised the study while the respondents were an integral part of the pilot thus creating the possibility of a bias with regards to responses obtained during the interviews. In order to mitigate this limitation, the implementers employed independent interviewers to conduct all interviews in addition to conducting the interviews while keeping the identity of the respondents confidential. Thus responses from the transcripts could not be traced to any particular individual.

In addition, the community reaction to the programme is a vital indication of how well the urban CHPS has been received by beneficiaries and this has not been assessed in this study. Thus further exploration of this area will be necessary in order to proffer a rounded assessment of the pilot. Furthermore, interviewing higher cadre individuals in the health care system may be vital in gaining insight into a practicable system wide approach to support the community based intervention.

## Conclusion

The establishment of the CHPS concept in the urban environment albeit challenging is an effort worth pursuing in a bid to narrow the gap caused by health inequalities in urban areas. The discussions highlight areas in which the CHOs have had to modify their services in order to meet the requirements of their clientele, as well as those which entail further adjustments. This study provides evidence that despite the constraints involved, it is possible to chart a course of action that will be acceptable to the community members and the healthcare work force alike by ensuring that vital elements of the programme are put into place to address the basic primary health care needs of peri-urban populations. Establishing an urban focused health policy will go a long way in ensuring that the challenges identified are addressed thus leading to an improved primary health care service delivery system tailored to the urban environment.

## Additional files

Additional file 1: IDI urban CHPS CHOs. Description of data: In-depth interview guide used for community health officers. (DOC 48 kb)

Additional file 2: IDI urban CHPS supervisors. Description of data: In-depth interview guide used for supervisors. (DOC 46 kb)

## Abbreviations

CCMm: Community Case Management of malaria
CHC: Community Health Committee
CHO: Community Health Officer
CHPS: Community-based Health Planning and Services
CWC: Child Welfare Clinic
GEHIP: Ghana Essential Health Intervention Programme
IDI: In-Depth Interview
IMNCI: Integrated Management of Neonatal and Childhood Illnesses
TEHIP: Tanzania Essential Health Intervention Programme
