# Supplementary material for: Implementing Community-based Health Planning and Services in impoverished urban communities: health workers’ perspective
Source: BMC Health Serv Res. 2018 Mar 20;18:186. doi: 10.1186/s12913-018-3005-1 (PMC5859666; doi:10.1186/s12913-018-3005-1)
Supplement: Supplementary file 1 — IDI urban CHPS CHOs. Description of data: In-depth interview guide used for community health officers. (DOC 48 kb) [file 12913_2018_3005_MOESM1_ESM.doc]

**IN DEPTH INTERVIEW GUIDE FOR CHOs**

*I am going to conduct an interview to gain an understanding of your experiences with Urban CHPS. The information we discuss will be kept confidential and will be used only for the purpose of this study, so please speak freely and share your honest opinions.*

*At this time, I will turn the audio recorder on.*

***First, I would like to get a sense of your general feeling about the project***

1. In your own words, can you please describe Urban CHPS?
2. From your perspective, what type of impact, if any, do you think urban CHPS has had in your community?

***I am now going to ask you a few questions about your experiences with the project***

***The first topic we will discuss is your work routine.***

a. Can you please describe your daily work routine.

1. What are some challenges you have faced while doing your job?
2. What changes have you made to your work plan to accommodate these issues?
3. How has your training been/not been adequate for the challenges you meet in the course of your work? (PROBE: Can you share some examples?)

***(2) Next I would like to learn more about the living accommodations for CHOs.***

1. Do you reside in the community that you serve?
2. [Only *ask if respondent answers no to question a]* What challenge does not living in your designated zone pose to your work routine and other related aspects of Urban CHPS?
3. [Only *ask if respondent answers no to question a]* How does this affect community acceptance of Urban CHPS?
4. What are the benefits of living in the community?

***(3) Let’s talk a bit about the service delivery aspect of Urban CHPS***

*Let’s begin with Home Visits and provision of Health Education*

1. Describe the services you deliver the most on home visits.
2. What are the areas of health education the community is most interested in?
3. Can you describe any challenges with home visitation and health education provision and how you handle these issues?
4. What type of impact, if any, do you think your home visits have?
5. What is your experience with meeting clients at home during your visits? (PROBE: Can you please explain?)
6. How does this affect your being able/not able to complete your required home visits schedule?

*I would now like to learn about problems related to mobility within the community*

1. What are some problems associated with moving around the community?
2. What can be done to alleviate (or lessen) these issues? (PROBE: Can you share an example?)

*Now I am going to list some activities. As I name each one, can you please highlight the important lessons learned from each one?*

- - - 1. Child Welfare/Immunization
      2. Malaria
      3. Antenatal and Postnatal Care
      4. Family Planning
      5. NCD- Referrals
      6. Diarrhea
      7. School Health Visits
      8. Community Health Education

*Next, I’d like you to elaborate on the challenging aspects of each of these activities as I name each one.*

1. Child Welfare/Immunization
2. Malaria
3. Antenatal and Postnatal Care
4. Family Planning
5. NCD- Referrals
6. Diarrhea
7. School Health Visits
8. Community Health Education

*Please elaborate on the aspects of each activity that can be changed to make it more successful. I will list each one again.*

- - 1. Child Welfare/Immunization
    2. Malaria
    3. Antenatal and Postnatal Care
    4. Family Planning
    5. NCD- Referrals
    6. Diarrhea
    7. School Health Visits
    8. Community Health Education

***(4) At this point, I would like to learn about the community reaction to CHPS***

1. How welcome or comfortable do you feel in the community where you work? (PROBE: Can you share any example?)
2. How does the community reaction to your presence vary by:
3. Gender
4. Marital status
5. Age
6. Religious affiliation
7. Educational qualifications
8. Ethnicity
   - - 1. ***Now, we are going to discuss about supervision.***
9. Could you begin by telling me how your job is supervised?
10. Can you elaborate on your opinion regarding if your supervisors meet your expectations
11. How engaged are your supervisors in the work you do?
    - - 1. ***Next, I would like to learn more about the logistics of your work as an Urban CHPS CHO***
12. On a typical day, what types of supplies do you carry with you?
13. Describe the challenges you face in having (in)sufficient supplies and carrying them around
14. What modifications would you like to see in the kind of supplies you carry? (PROBE: Any more?)
    - - 1. ***Let’s move forward and talk about how you are working with the volunteers and health committees.***
15. What is the role of volunteers in the Urban CHPS programme?
16. Please describe the role of the Community Health Committee and CHC members?
17. How often do you meet with volunteers?
18. How often do you meet with CHC members?
19. Describe any challenges you have experienced in dealing with volunteers and CHC members.
20. How do you handle these challenges?
    - - 1. ***What modifications or changes do you think will make Urban CHPS more successful?***
        2. ***We have now come to the end of our interview. Is there anything else you would like to bring up that we did not cover during our discussion?***

*(If yes, continue discussion and record response)*

*(If no, say thank you, end interview and shut of audio recorder)*
