# Supplementary material for: Implementing Community-based Health Planning and Services in impoverished urban communities: health workers’ perspective
Source: BMC Health Serv Res. 2018 Mar 20;18:186. doi: 10.1186/s12913-018-3005-1 (PMC5859666; doi:10.1186/s12913-018-3005-1)
Supplement: Supplementary file 2 — IDI urban CHPS supervisors. Description of data: In-depth interview guide used for supervisors. (DOC 46 kb) [file 12913_2018_3005_MOESM2_ESM.doc]

**IN-DEPTH INTERVIEW GUIDE FOR THE SUPERVISORS**

*I am going to conduct a short interview to gain an understanding of your experiences with Urban CHPS. The information we will discuss will be kept confidential and will be used only for the purpose of this study, so please speak freely and share your honest opinions.*

*At this time, I will turn the audio recorder on.*

*First, I would like to gain a sense of your general impressions of the Urban CHPS project.*

1. In your own words, please describe Urban CHPS
2. What impact do you think that CHPS has made in the communities?

*At this point I would like to talk a bit about your experiences with the project*

***A. I would first like to discuss with you aspects related to service delivery.***

*Specifically, let’s talk about home visits and the provision of health education.*

1. Please describe what services are generally provided during home visits
2. From your perspective, what areas of health education are communities most interested in?
3. Describe any challenges CHOs have encountered when conducting home visits or providing health education. In what ways did they cope with these issues?
4. Discuss any issues encountered while trying to meet clients during home visits. How did this impact the ability to complete home visit scheduling requirements?
5. Overall, what did you think was the greatest impact, if any, of the home visits?

*Now I am going to ask you to describe overall trends you have observed in service utilization in the communities. As I list each one, please elaborate, if you are able, on the proposed topics:*

- - 1. Child Welfare/Immunization
    2. Malaria
    3. Antenatal and Postnatal Care
    4. Family Planning
    5. NCD- Referrals
    6. Diarrhea
    7. School Health Visits
    8. Community Health Education
       - 1. ***Now I am interested in learning more about reactions of CHPS within the community.***

1. What reaction, if any, did CHOs receive from community members?
2. Did you feel that communities did or did not welcome your presence working in their communities? (PROBE: How so?)
3. What activities were held in the communities to enhance awareness or knowledge of CHPS services? (PROBE: How often?)
4. From your perspective, how did community members react based on these following characteristics:
   1. Their gender (PROBE: How so?)
   2. Their marital status (PROBE: How so?)
   3. Their age (PROBE: How so?)
   4. Their religious affiliation (PROBE: How so?)
   5. Their educational status (PROBE: How so?)
   6. Their ethnicity (PROBE: How so?)
      - - 1. ***Now I would like to gather information on how you partake in the supervision and monitoring of the Urban CHPS project.***

*1. First, let’s discuss aspects related to supervision.*

1. Please describe how you conduct supervision for the Urban CHPS program? (PROBE: How often does this happen?)
2. What are some challenges you have faced while providing this supervision?
3. How have you coped with these challenges? (PROBE: Do you have an example?)
4. In what ways, if any, do you think that the CHO trainings are inadequate (PROBE: In what ways could their trainings be improved?)
5. *Now I would like to discuss the overall monitoring of the CHOs by the district and regional levels.*
6. Are monitoring activities currently being conducted by the regional health administration? (PROBE: In what ways is this occurring? How often does this occur?)
7. From the district level, is any routine monitoring of activities tacking place? (PROBE: In what was is this occurring? How often does this take place?)
   - - - 1. ***I would now like to learn more about the logistics involved in the delivery of the Urban CHPS program.***
8. From your perspective, in what ways could CHOs be better equipped? (PROBE: Sufficient supplies to carry out activities?)
9. I would now like to learn more about your thoughts on the organizational capacity of the Urban CHPS project: adequacy of facilities, supplies, and workforce, sustainability of these operations (PROBE: Why do you think that it is/is not sustainable?)
   - - - 1. ***At this point I would like to talk a bit about your work with the volunteers and health committees***
10. In your own words, please describe the role of community volunteers. How often do you meet with community volunteers? Please describe these meetings.
11. What do you think is the role of Community Health Committees? How often do you meet with CHC members? Please describe these meetings.
12. Please describe any challenges encountered while working with community volunteers? What about community health committees?
13. How have you handled these challenges?
    - - - 1. **Now before we finish up, is there anything else about your experience with Urban CHPS that you think is important to the future of the project that we have not discussed?**

*(If yes, continue discussion and record response)*

*(If no, say thank you, end interview and shut of audio recorder)*
